# Physiological and analgesic effects of continuous-rate infusion of morphine, butorphanol, tramadol or methadone in horses with lipopolysaccharide (LPS)-induced carpal synovitis

**DOI:** 10.1186/s12917-014-0299-z

**Published:** 2014-12-21

**Authors:** Adriano B Carregaro, Gabrielle C Freitas, Martina H Ribeiro, Nathalia V Xavier, Renata GS Dória

**Affiliations:** Department of Veterinary Medicine, University of São Paulo, Duque de Caxias Norte ave 225, Pirassununga, ZIP 13635-900 SP Brazil; College of Veterinary Medicine, Federal University of South Border, Realeza, PR Brazil

**Keywords:** Opioids, Analgesia, Inflammatory pain

## Abstract

**Background:**

Continuous-rate infusion (CRI) of drugs results in more stable plasma drug concentrations than administration of intermittent boluses, thus providing greater stability of physiological parameters. The aim of this study was to evaluate the physiologic and analgesic effects of the administration of morphine, butorphanol, tramadol or methadone by CRI in horses with induced synovitis of the radiocarpal joint.

**Results:**

Increased values of cardiorespiratory parameters and body temperature were observed in all groups after initiation of opioid administration, and these increases were sustained throughout the CRI period. Morphine, butorphanol and methadone each caused a reduction in gut sounds, and this effect was greatest in animals that received morphine. Administration of morphine or methadone reduced the degree of lameness after the end of intravenous infusion. Administration of tramadol did not alter the degree of lameness in the animals.

**Conclusions:**

CRI of morphine or methadone, but not butorphanol or tramadol, provided analgesia in horses with carpal synovitis. All of these opioids increased cardiovascular and respiratory parameters and reduced gut sounds during CRI.

## Background

Despite the analgesia provided by opioids, in pain free horses they may procude dose-dependent central nervous system (CNS) excitation [1-3], increased spontaneous locomotor activity [4] and reduced intestinal motility [5,6]. Ataxia, shivering and restlessness have been reported in horses that received a single IV injection of 0.1 mg/kg butorphanol [7,8]. The same side effects have been reported for IV injection of 0.5 mg/kg methadone [9]. Tramadol also elicited undesirable effects, especially muscle twitching and head nodding, after 1.5 or 3 mg/kg rapid IV administration [10]. Clinically relevant doses of morphine (0.05 and 0.1 mg/kg) yielded minimal and short-term cardiorespiratory, gastrointestinal and behavioural effects, but analgesia was only achieved for a short period of time in healthy conscious horses with no signs of pain [11]. The dose, route, and duration of the opioid treatment likely influenced whether adverse effects developed and the severity of those effects [6,12].

The use of CRI minimises changes in plasma concentrations, which are frequent in intermittent bolus administrations. Side effects may be less likely to occur during CRI because the total dose of the drug over time is lower than the total administered boluses [7,13]. CRI of morphine during inhalation anaesthesia did not promote any haemodynamic or pulmonary changes [14], and it provided a shorter, better-quality anaesthetic recovery compared to the no-morphine group [14,15]. CRI of butorphanol caused a lower incidence of behavioural and gastrointestinal alterations and lower plasma cortisol levels than the saline group in horses after celiotomy [16]. Moreover, those horses displayed less weight loss while hospitalised and recovered quickly.

However, some studies report no advantages for CRI of opioids in horses. Bettschart-Wolfensberger et al. [17] observed no isoflurane-sparing effect with CRI of butorphanol, and animals that had received this opioid showed longer times to extubation than receiving only medetomidine [17]. Villalba et al. [18] reported that morphine did not improve the isoflurane-sparing effect in horses undergoing CRI of ketamine plus lidocaine [18]. In another study, CRI of morphine showed no advantages over CRI of dexmedetomidine in isoflurane-anaesthetised horses undergoing elective surgery [19]. Furthermore, horses treated with morphine developed post-operative colic and excitatory behaviour. To the authors’ knowledge, no studies to date have determined whether a bolus dose followed by CRI of tramadol or methadone provides analgesia in horses or whether it has an influence on horse physiology.

The aim of this study was to evaluate the physiological and analgesic effects of the administration of morphine, butorphanol, tramadol or methadone by CRI in horses subjected to synovitis induced by injection of *Escherichia coli* lipopolysaccharide (LPS) into the radiocarpal joint.

## Methods

This study was approved by the University of São Paulo Animal Care Committee, under protocol number 2012.1.106.74.4. Fourteen adult horses from the University of São Paulo farm were used in the experiment, 9 females and 5 males weighing between 350 and 500 kg, and they were considered clinically healthy as assessed by clinical examination and laboratory tests (complete blood count, and biochemical profile). The study consisted of four experimental groups, with each animal participating in two out of the four conditions. The washout period between the treatments was at least one month.

For induction of synovitis, 0.5 ng of LPS of the *Escherichia coli* strain 055:B5 (*E. coli* 055:B5; Sigma Chemical Co., St Louis, MO, USA) was aseptically administered into the right or left radiocarpal joint according to the experimental design. The LPS was diluted in phosphate-buffered saline (pH 7.4) to a final volume of 1 mL for each administration. After clinical examination of the physiological parameters (baseline), the animals were sedated with 1 mg/kg xylazine IV (Sedazine - Schering-Plough, Rio de Janeiro, RJ, Brazil). Once sedated, the horses were given an intra-articular administration of LPS [20].

Only two of the horses were submitted to a trial study without any opioid treatment. The trial study revealed that within three hours post-induction of synovitis, there was already an evident degree of lameness (3 or 4). Accordingly, three hours after LPS injection, jugular venipuncture was performed with a 14G catheter and used for the administration of one of four CRI treatments: morphine (Mo, bolus of 0.15 mg/kg followed by CRI of 0.1 mg/kg/h morphine; Dimorf 10 mg/mL, Cristalia Prod Quim Farm Ltda, Itapira, SP, Brazil), butorphanol (B, bolus of 25 μg/kg followed by CRI of 25 μg/kg/h butorphanol; Torbugesic, Fort Dodge Saude Animal Ltda, Campinas, SP, Brazil), tramadol (T, bolus of 1.5 mg/kg followed by CRI of 1 mg/kg/h tramadol; Tramadon, Cristalia Ltda, Itapira, SP, Brazil) or methadone (Me, bolus of 0.25 mg/kg followed by CRI of 0.25 mg/kg/h methadone; Mytedon 10 mg/mL, Cristalia Ltda, Itapira, SP, Brazil). All treatments were assigned in a randomised manner. The bolus and infusions were administered using a syringe infusion pump (DigiPump SR8x, Digicare Animal Health, Boynton Beach, FL, USA) and maintained for three hours, where the volumes were standardised with a bolus of 17 mL/10 min and CRI of 50 mL/h such that the observers were blinded to the treatment.

Physiological parameters and analgesia were evaluated before sedation (baseline) and 1.5, 3, 4, 5, 6, 7, 8, 9, 10, 12, 14, 16 and 24 hours after induction of synovitis. Heart rate (HR) was determined with a stethoscope between the 3rd and 6th intercostal space and was measured in beats per minute (bpm). Systolic arterial pressure (SAP) in mmHg was measured non-invasively using an ultrasonic Doppler (Doppler, model 841 A, Parks Medical Electronics) with the transducer positioned over the coccygeal artery at the base of the tail. The measurements were taken with the cuff positioned cranially to the transducer such that the cuff’s width was half the diameter of the tail [21]. The respiratory rate (RR) was measured by manually counting chest movements per minute (mpm), and the core body temperature (T°C) was measured with a flexible digital thermometer (Pro-Check, ONBO Electronics, USA) inserted approximately 3 cm into the rectum.

Gut sounds were evaluated by abdominal auscultation, dividing the right lateral flank into two portions and determining the opening of the ileocecal valve (dorsal portion) and the right ventral colon (ventral portion). The same procedure was performed on the left side. Each quadrant was auscultated for one minute, with an interval of a few seconds between quadrants. Scores of 0 to 4 were noted and later summed, resulting in values between 0 and 16 [1].

To assess lameness, the horses were recorded while being led at a walk and at a trot in a straight line (50 metres) on a hard surface. The images were analysed in a random order, after the end of all treatments, by an experienced observer (Dr. Dória) who was unaware of the treatments used and the time of each measurement. The lameness was scored as follows: 0, absence of visible lameness; 1, discrete asymmetry, occasionally inconsistent; 2, visible lameness, rarely inconsistent; 3, visible lameness at all times; and 4, no weight bearing [22]. The highest scores for walking or trotting were considered for statistical purposes. At each evaluation point, physiological parameters were measured before each lameness assessment. CRI of the opioid was interrupted for only two minutes at 3, 4 and 5 hours so that the animal’s walking and trotting could be filmed.

Rescue analgesia was administered to animals that showed after each evaluation, a lameness score of 3 or 4 and that did not show any improvement of gait, or that showed any signs of moderate to severe pain at 6 hours (end of CRI). This was evaluated by one experienced observer (Dr. Carregaro) who was unaware of all treatments. For rescue analgesia, 0.1 mg/kg morphine and 1 mg/kg flunixin meglumine were administered IV. The animals were then continuously monitored to check the improvement of their clinical status. All animals were given 1 mg/kg flunixin meglumine IV once a day for three days after the evaluation period.

Statistical analysis was conducted using GraphPad Prism (GraphPad Software, San Diego, CA, USA). The parametric variables HR, SAP, RR and T°C were evaluated by analysis of variance (ANOVA) with Dunnett’s post-test for comparisons of means within each group in relation to baseline. Comparisons between groups at each time were performed with one-way ANOVA followed by Tukey’s test. The Friedman test was utilised for the non-parametric variables gut sounds and lameness. The parametric results were expressed as mean ± standard deviation, and the non-parametric results were expressed as the median and interquartile range. Differences were considered significant when P < 0.05.

## Results

At 3 hours, all animals showed signs of pain, such as lowered head, reluctance to move, and a peculiar facial expression [23] with stiffly backward ears, orbital tightening, strained nostrils and flattening of the profile. During CRI, signs of restlessness were observed in two animals in the B group, two in the Mo group and one in the Me group. These animals showed continuous head nodding, digging, and shifting of limbs. In contrast, the other animals in the same groups remained calm during CRI. No animal in the T group showed excitation, but all animals were unable to stand on the affected leg and shifted weight to their hind legs. Skin twitching was also observed in four animals in the T group and three in the Me group, particularly in the upper regions of the forelimbs and the dorsolateral area of the thorax and abdomen, especially during the first 30 minutes after bolus injection.

**Figure 1:**
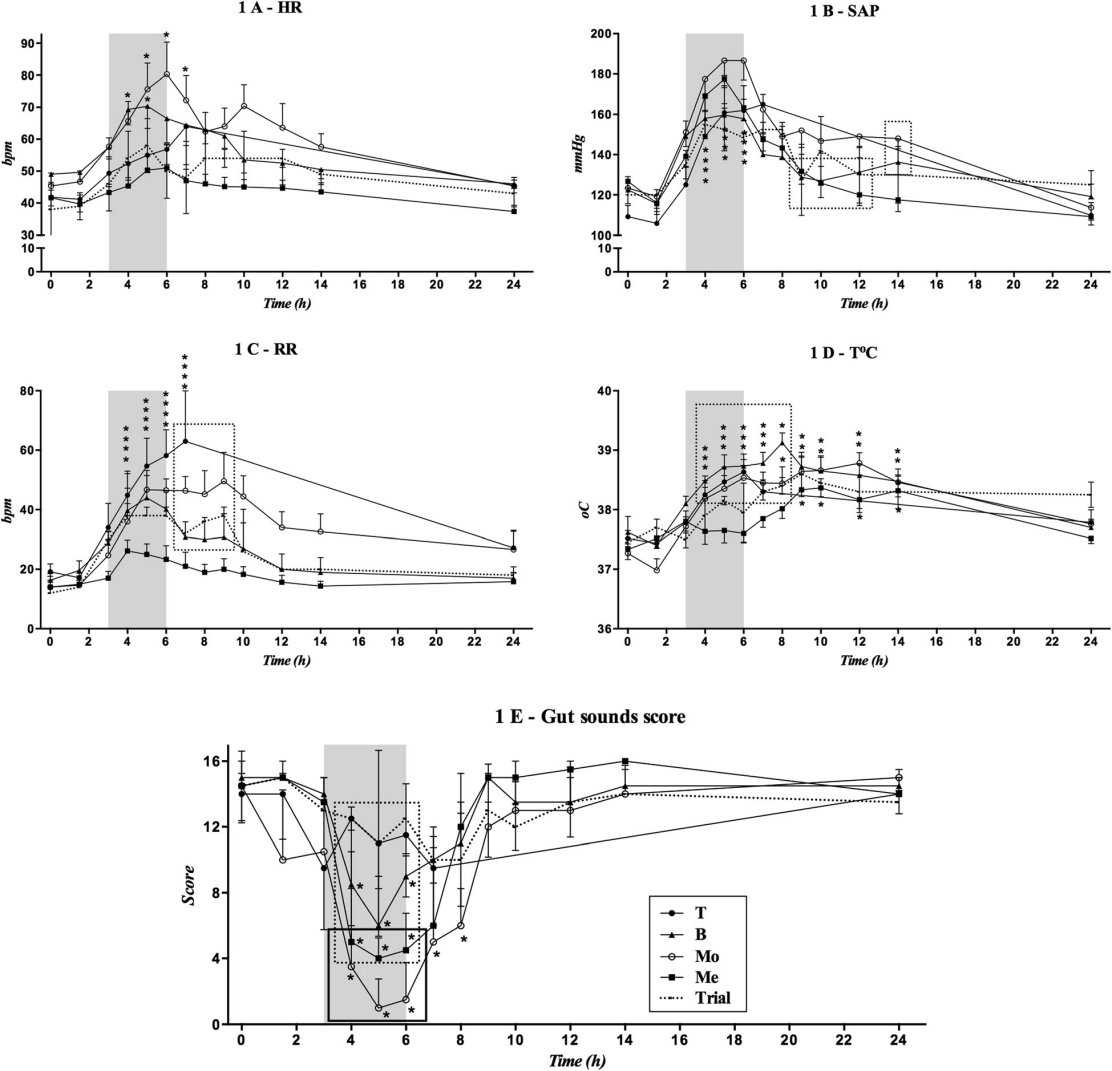
**Effects of 0.25 mg/kg + 0.25 mg/kg/h methadone (Me), 0.15 mg/kg + 0.1 mg/kg/h morphine (Mo), 25 μg/kg + 25 μg/kg/h butorphanol (B), and 1.5 mg/kg + 1 mg/kg/h tramadol (T) on heart rate (HR – 1A; bpm – beats/minute), systolic arterial pressure (SAP – 1B; mmHg), respiratory rate (RR – 1C; bpm – breaths/minute), core body temperature (T°C – 1D; °C), and gut sounds score (1E) of horses (n = 6) submitted to the experimental synovitis model.** Values are reported as the mean ± SEM. Treatments were at 3 to 6 hours (in grey). The pilot study was conducted with 2 horses that received no opioid treatment (--- Trial). *Within each group, values differ significantly (P < 0.05) from the baseline value (0 minute). Values at the same time point in squares/rectangles indicate no significant difference (P < 0.05).

All groups showed an increase in cardiorespiratory parameters after the beginning of the treatments, with a marked increase in HR in animals of the B and Mo groups and in SAP in all groups. After the end of the treatments (6 hours), the parameters gradually returned to the baseline values. Temperature increased in the Mo, B and T groups; it increased between 4 hours and 7 hours in the T group (the last time point evaluated in this group) and to 14 hours in the Mo, Me and B groups (Figure 1). There was a marked decline in gut sounds in the Mo group at 4 hours, and this effect persisted up to 8 hours. Additionally, reduced gut sounds were observed in the Me and B groups during the infusion period. This effect was not observed in the T group (Figure 1). Notably, all animals in the T group defaecated at least once during the three hours in the stock. Defaecation was also observed for two animals in the B group and for none in the Mo and Me groups.

Lameness was moderate at 1.5 hours and severe at 3 hours after LPS injection [22]. After 3 hours, and as in the pilot study, practically all animals showed the maximal degree of lameness except for two animals in the T group and one animal in the Me group, which showed a score of 3. During CRI, gradual improvement was noted in the Mo and Me groups. Moreover, both groups demonstrated better lameness scores, starting at 6 hours, whereas the animals in the B group only showed improvement starting at 8 hours. No improvement was observed in the animals in the T group (Table 1).

**Table 1:** **Degree of lameness of horses (n = 6) submitted to the experimental synovitis model and treated with 0.25 mg/kg + 0.25 mg/kg/h methadone (Me), 0.15 mg/kg + 0.1 mg/kg/h morphine (Mo), 25 μg/kg + 25 μg/kg/h butorphanol (B), and 1.5 mg/kg + 1 mg/kg/h tramadol (T)**

		**Time (hours)**												
					**Continuous rate infusion**									
		**0**	**1.5**	**3**	**4**	**5**	**6**	**7**	**8**	**9**	**10**	**12**	**14**	**24**
**Lameness score**	Me	0 (0–0)	1.5 (0.5-2)*	4 (3.5-4)	3.5 (3–4)	2 (2–2.5)a	1 (1–1.5)*a	1.5 (0.5-2)*a	1.5 (1–2)*	1 (1–1.5)*	1 (1–1.5)*	1.5 (1–2)*	1 (1–1.5)*	0 (0–0)*
	Mo	0 (0–0)	3 (2–3)	4 (4–4)	4 (3.5-4)	3 (3–4)ab	2 (2–3)*a	2.5 (2–3)*a	2 (2–3)*	2 (2–3)*	2 (2–2.7)*	2 (1.2-2.7)*	2 (1.2-2.7)*	0 (0–1)*
	B	0 (0–0)	2 (2–3)*	4 (4–4)	4 (4–4)	4 (4–4)ab	4 (4–4)b	3 (2.5-3)a	2 (1.5-3)*	2 (1–3)*	2 (1–3)*	1.5 (0–2.2)*	1.5 (0–2)*	0 (0–0)*
	T	0 (0–0)	3 (2.7-3)	4 (3–4)	4 (3.7-4)	4 (3–4)b	4 (3–4)b	3.5 (3–4)b	--	--	--	--	--	0 (0–0)*
	Trial	0 (0–0)	3 (3–3)	4 (4–4)	4 (4–4)	4 (4–4)	4 (4–4)	4 (4–4)	4 (4–4)	3.5 (3–4)	4 (4–4)	3 (3–3)	3 (3–3)	1.5 (1–2)

The pilot study was conducted with 2 horses that received no opioid treatment (Trial). Values reflect the median and interquartile range.

Continuous rate infusion was performed between 3 and 6 hours (grey).

*Difference relative to 3-hour baseline.

Within a time point, values with different letters differ significantly.

All animals in the T group required rescue analgesia at the end of the treatment, among which four were rescued at 6 hours and two at 7 hours. Two animals in the B group were rescued at 6 hours, and only one animal in the Mo group was rescued at the same time. None of the animals in the Me group required rescue analgesia. All rescued animals showed improvement that was evident approximately 15 minutes after treatment. After an hour, lameness was minimal or absent, and the physiologic parameters were close to normal for the species. The data obtained after rescue are not shown and were not considered for statistical evaluation. All animals, rescued or not, showed normal mobility at 24 hours post LPS injection.

## Discussion

Our results showed a marked reduction in the lameness of animals treated with morphine or methadone, demonstrating that CRI treatments were reasonably effective against experimentally induced inflammatory joint pain. Moreover, there was no need for rescue analgesia in animals treated with methadone. Methadone has a mu-receptor affinity similar to the affinity of morphine [24], but one should consider that the dose of methadone was twice as high as the morphine dose and must have contributed to the higher analgesic effect. Information about the analgesic effects of methadone in horses is limited, but recently, it was demonstrated that a single IV dose of 0.2 mg/kg did not produce a consistent antinociceptive effect to thermal and electrical stimuli. The administration of 0.5 mg/kg induced short-acting antinociception but may have been limited due to adverse behavioural effects and ataxia in horses [9].

To the authors’ knowledge, there are no published studies in which only morphine administered as a CRI was used as a unique treatment in clinical studies of horses. The available studies report the antinociceptive effects of morphine in inhalation anaesthesia [14] or combined with other analgesics [18,25,26]. The morphine dose chosen for treatment was the same as that used in the studies of Clark et al., who showed that horses receiving morphine during the anaesthetic period tended to receive fewer and lower doses of additional anaesthetic drugs in the intra- and postoperative periods [14] and to recover better from the anaesthesia [15] than control animals.

The bolus dose and CRI of butorphanol were in accordance with those used by other authors [16,27,28]. However, it should be noted that there was no standardisation of the dose or of the time of infusion. Even less information is available for tramadol and methadone in horses, which still have not been tested using CRI in this species. The choice of the tramadol doses was based on Grond & Sablotzki (2004) [29], who noted that the potency was approximately 10% of that of morphine. As no studies using methadone administered by CRI in horses exist, the bolus and CRI rate were established based on Linardi et al. [30], who showed that a single 0.15 mg/kg dose of methadone was insufficient to promote analgesia, and based on Oliveira et al. [9], who studied two doses of methadone (0.2 and 0.5 mg/kg) and observed that the higher dose promoted intense ataxia.

One study showed that morphine failed to promote analgesia when administered in a single dose to treat thermal and electrical noxious stimuli [11]. A single dose (0.25 mg/kg) of morphine did not cause an appreciable change in the minimum alveolar concentration of isoflurane, and it was not indicated for routine clinical use as an adjuvant to isoflurane anaesthesia in horses [31]. However, both studies were performed with an electrical and/or thermal stimulus, which may not represent inflammatory pain. As noted for CRI, Clark et al. [14] suggested that a dose of 0.15 mg/kg followed by 0.1 mg/kg/h of morphine appeared to produce analgesia in animals undergoing surgery. However, this study did not state how long the infusion was administered; this parameter could have influenced the results, as the results obtained here only demonstrated efficacy after 3 hours of infusion.

CRI of butorphanol decreased lameness only at 8 hours. Nonetheless, the results should be interpreted with caution, as two animals required rescue analgesia (Dr. Carregaro’s evaluation), and the lameness scores were higher than those after morphine and methadone treatment, demonstrating a clinical difference. Furthermore, if the lameness scores indicated by Dr. Dória were considered for establishing rescue analgesia, not two but rather five horses should have received it, demonstrating the ineffectiveness of CRI with butorphanol. Although the observers were experienced in the evaluation of lameness in horses, it should be noted that variations can occur, mainly because of the use of subjective scales [32], thereby resulting in differences among the authors’ scores.

We were not able to demonstrate analgesia with tramadol. The analgesic action of tramadol in horses has been studied in the past few years, but only one study reports transient analgesia in horses with pain caused by chronic laminitis. In that study, tramadol alone (orally at 5 mg/kg every 12 h) provided limited pain relief, and it was only effective when combined with CRI of ketamine [33].

The lack of analgesia may have resulted from the biotransformation of tramadol in the horses. In horses, tramadol likely undergoes hepatic biotransformation [34] and also produces at least five metabolites [35,36]. The main active metabolite of tramadol, O-desmethyltramadol, acts on opioid receptors. N-desmethyltramadol is the inactive metabolite [34]. Although it has been suggested that N-desmethyltramadol is the major metabolite produced [34,35], recent studies have demonstrated that horses are very efficient in metabolising tramadol to O-desmethyltramadol (phase I reaction). However, the latter metabolite is rapidly and extensively conjugated with glucuronic acid (phase II reaction) such that the fraction of nonconjugated O-desmethyltramadol in plasma is truly low [10,37,38]. In light of this biotransformation, we proposed a treatment of CRI with tramadol in horses. Thus, it was speculated that the accumulation of O-desmethyltramadol, combined with other pathways of action for tramadol, could provide analgesia, but the results showed no effect for tramadol. Another possibility is that the lack of analgesia may have resulted from the dose chosen, which may have been sub-therapeutic.

Prior to CRI opioid administration, there was a slight increase in cardiorespiratory parameters in all groups, indicating the physiological effects of the pain model that was used. Regarding morphine and butorphanol, some papers report an absence of cardiovascular alterations upon CRI administration to horses. The use of butorphanol was not associated with cardiovascular changes, even during a 24-hour infusion [7]. Another study also showed that morphine did not induce cardiovascular changes in horses, but the animals were under inhalation anaesthesia [14]. Additionally, restlessness may have induced the increase in these parameters in the Mo, B and Me groups. Despite the lack of differences among the groups during CRI, higher SAP values were observed in all animals; they markedly decreased after the infusions, indicating that the opioids increased the SAP values [24,27,39].

We also suggest that the physiologic changes in the T group may have resulted from the synovitis model, mainly considering the ineffectiveness of tramadol. This idea is reinforced by the few studies that report no substantial changes in cardiovascular parameters, even with such higher doses as 2 and 5 mg/kg of tramadol [40-42]. In relation to the influence of pain and opioids on cardiovascular parameters, the inclusion of a real control group, i.e., a synovitis-only group, could have addressed this concern. However, for ethical reasons, such a group was not used.

Despite the reduced doses of opioids used during the CRI in this study, reduced gut sounds were still observed from the start of the infusion of butorphanol, methadone and especially of morphine. Thus, not only the pure agonists but also butorphanol caused a reduction in gut sounds. In other studies, gut sounds did not vary significantly either during or after CRI of butorphanol, but the number of faecal piles passed during the 24-hour treatment period was reduced [7,16]. This finding should be carefully considered because of the strong call for the use of butorphanol in horses, as this drug theoretically has little influence on gut sounds. No changes in gut sounds were observed in the T group, corroborating other authors [33,40]. In contrast, another study reported a case of mild colic in one horse after six consecutive doses of 10 mg/kg tramadol. Thus, gastrointestinal depression with tramadol may not be common, but it is possible.

Hyperthermia was observed in the Mo, B and T groups and could have occurred as a result of the direct LPS action on the thermoregulatory centre and through the synthesis and secretion of endogenous pyrogens from neutrophils and mononuclear phagocytes, which are released as a result of joint inflammation [43]. However, given that the Me group animals did not show hyperthermia, methadone may also have anti-inflammatory properties [44].

Some limitations should be considered in the present study. The inclusion of a control group could show the influence of pain and opioid on cardiovascular parameters. In addition, if a dose–response study had been performed, we could have drawn a proper comparison between the analgesic effects of morphine and methadone and even examined the reason why tramadol and butorphanol were not effective as administered in this study. Furthermore, it is important to point out that the dose of methadone was twice as high as the morphine dose, which must have contributed to the superior analgesic effect of methadone over morphine.

## Conclusions

Infusions of morphine and methadone reduced the pain of horses with carpal synovitis. However, it is suggested that CRI of these opioids in horses can increase cardiovascular and respiratory variables in addition to causing reduced gut sounds.

## Abbreviations

CRI: Continuous rate infusion
LPS: Lipopolysaccharide
IV: Intravenous
Mo: Morphine group
B: Butorphanol group
T: Tramadol group
Me: Methadone group
HR: Heart rate
SAP: Systolic arterial pressure
RR: Respiratory rate
T°C: Core body temperature
